# First-principles investigations of metal–semiconductor MoSH@MoS_2_ van der Waals heterostructures

**DOI:** 10.1039/d3na00465a

**Published:** 2023-08-17

**Authors:** Son-Tung Nguyen, Cuong Q. Nguyen, Nguyen N. Hieu, Huynh V. Phuc, Chuong V. Nguyen

**Affiliations:** a Faculty of Electrical Engineering, Hanoi University of Industry Hanoi 100000 Vietnam nguyensontung@haui.edu.vn; b Institute of Research and Development, Duy Tan University Da Nang 550000 Vietnam nguyenquangcuong3@duytan.edu.vn; c Faculty of Natural Sciences, Duy Tan University Da Nang 550000 Vietnam; d Division of Theoretical Physics, Dong Thap University Cao Lanh 870000 Vietnam; e Department of Materials Science and Engineering, Le Quy Don Technical University Hanoi Vietnam

## Abstract

Two-dimensional (2D) metal–semiconductor heterostructures play a critical role in the development of modern electronics technology, offering a platform for tailored electronic behavior and enhanced device performance. Herein, we construct a novel 2D metal–semiconductor MoSH@MoS_2_ heterostructure and investigate its structures, electronic properties and contact characteristics using first-principles investigations. We find that the MoSH@MoS_2_ heterostructure exhibits a p-type Schottky contact, where the specific Schottky barrier height varies depending on the stacking configurations employed. Furthermore, the MoSH@MoS_2_ heterostructures possess low tunneling probabilities, indicating a relatively low electron transparency across all the patterns of the MoSH@MoS_2_ heterostructures. Interestingly, by modulating the electric field, it is possible to modify the Schottky barriers and achieve a transformation from a p-type Schottky contact into an n-type Schottky contact. Our findings pave the way for the development of advanced electronics technology based on metal–semiconductor MoSH@MoS_2_ heterostructures with enhanced tunability and versatility.

## Abstract 2

Two-dimensional (2D) metal–semiconductor heterostructures play a critical role in the development of modern electronics technology, offering a platform for tailored electronic behavior and enhanced device performance.

## Graphical abstract

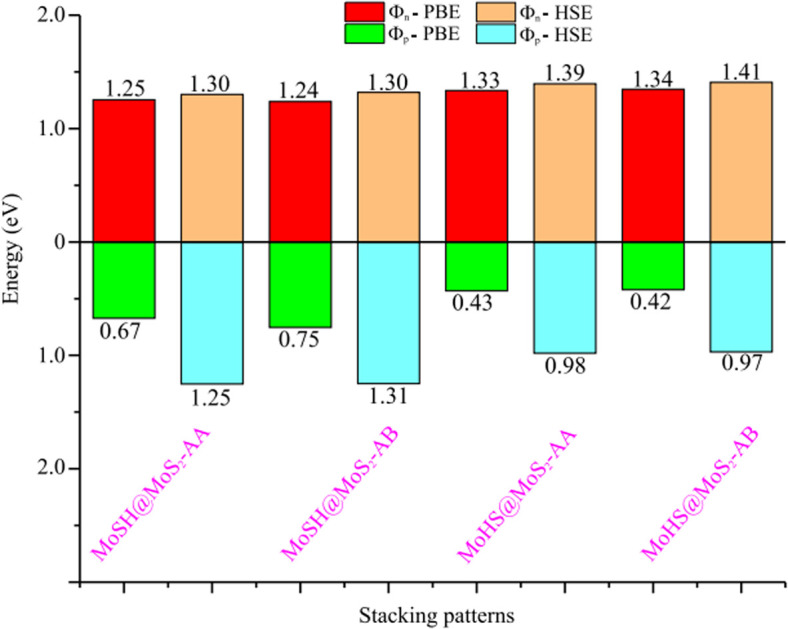

## 1. Introduction

The emergence of two-dimensional (2D) materials^1–3^ has brought about a paradigm shift in our comprehension of material properties and their potential applications in electronic devices. These atomically thin materials have captivated researchers by virtue of their remarkable electronic, optical, and mechanical attributes, paving the way for groundbreaking technological advancements. 2D materials possess exceptional properties that distinguish them from their bulk counterparts. The confinement of electrons within a single layer leads to quantum confinement effects, which give rise to a multitude of fascinating phenomena. These materials exhibit novel electronic band structures, such as the presence of Dirac cones in graphene^4^ or the emergence of bandgaps in transition metal dichalcogenides (TMDs) such as MoS_2_ (ref. 5) or controllable catalytic performance by transition metal-doping.^6–9^ The absence of dangling bonds at the edges of 2D materials grants them exceptional stability, making them ideal for various applications, including electronic and optoelectronic devices,^10,11^ energy storage and conversion systems^12,13^ and gas sensors.^14,15^

Furthermore, an important advantage of 2D materials is their ability to form vertically stacked van der Waals (vdW) heterostructures, exhibit unique properties and enable the creation of functional materials.^1,16–18^ These vdW heterostructures offer a versatile and novel architecture for the development of ultracompact devices. The integration of 2D vdW heterostructures presents a highly versatile approach that capitalizes on the unique properties of individual 2D materials. Recently, metal–semiconductor heterostructures have played a critical role in the development of modern electronic devices.^19,20^ These heterostructures form the basis of device architectures and are essential for their proper functioning. Recently, there has been significant progress in the synthesis and prediction of 2D metal–semiconductor heterostructures, such as graphene/TMDs,^21–24^ NbSe_2_/WSe_2_,^25^ NbS_2_/MoS_2_ (ref. 26) and so forth.^27–30^

Recently, a groundbreaking experimental synthesis of a novel metallic monolayer, known as MoSH, has been reported.^31^ Similar to graphene, the Janus MoSH monolayer exhibits metallic properties and possesses a high intrinsic carrier concentration. This intrinsic metallic character imparts exceptional electrical conductivity, thereby presenting exciting possibilities for its application in electronic and optoelectronic devices. Moreover, the Janus MoSH monolayer exhibits intrinsic superconductivity.^32,33^ This remarkable property further underscores the potential of Janus MoSH for enabling superconducting technologies. Furthermore, He *et al.* explored the electronic, mechanical, and piezoelectric characteristics of the Janus MoSH monolayer.^34^ Their findings suggest that Janus MoSH possesses desirable ductility and exhibits high piezoelectric coefficients, thus positioning it as a promising candidate for efficient sensors and piezoelectric components. In recent years, there has been a growing interest in exploring the combination of Janus MoSH with other two-dimensional (2D) semiconductors, such as MoSH/WSi_2_N_4_^35^ and MoSH/MoSi_2_N_4_.^36^

However, the combination between metallic Janus MoSH and semiconducting MoS_2_ monolayers has not yet been extensively explored or reported. While there is significant interest and ongoing research in the field of metal–semiconductor heterostructures, the specific combination of Janus MoSH with MoS_2_ remains a relatively unexplored area. The integration of these materials could potentially yield novel electronic and optoelectronic functionalities, making it an area of interest for researchers working in the field of metal–semiconductor heterostructures and 2D material combinations. Therefore, in this work, we constructed a novel metal–semiconductor MoSH@MoS_2_ heterostructure and investigated its structures, electronic properties, and potential applications. Our findings have implications for the fundamental understanding of metal–semiconductor heterostructures and provide valuable insights for the development of advanced electronic technologies.

## 2. Computational methods

In this work, we employed first-principles calculations to predict the metal–semiconductor MoSH@MoS_2_ heterostructures and investigate their structures, electronic properties and contact characteristics, as well as the effects of applied electric fields. The simulation package Quantum Espresso^37,38^ was used to calculate all the properties of materials. The exchange–correlation energy was described using the Perdew–Burke–Ernzerhof (PBE) functional within the generalized gradient approximation.^39^ Furthermore, we adopted the projector augmented wave (PAW) method^40^ to accurately account for the electron–ion interaction. In all our calculations, we utilized a cut-off energy of 510 eV and employed a Monkhorst–Pack *k*-point grid of 9 × 9 × 1. These computational parameters were carefully chosen to ensure accurate and reliable results for our investigations. Additionally, to describe the weak interactions existing in layered materials, we employed Grimme's DFT-D3 method.^41^ We incorporated a vacuum space of 25 Å along the *z*-direction to prevent interactions between periodic images and ensure an accurate representation of the isolated MoSH@MoS_2_ heterostructures. To ensure the convergence of our calculations, we set the convergence criteria for the total energy and force to 10^−6^ eV and 0.01 eV Å^−1^, respectively. In order to obtain a more accurate band gap for materials, we employed the state-of-the-art hybrid functional HSE06 (ref. 42) to calculate the electronic properties of the MoSH@MoS_2_ heterostructures. Additionally, it should be noted that the intrinsic dipole has a crucial role in 2D Janus materials, particularly in semiconducting Janus materials such as Cr_2_NY (Y = P, As, Sb)^43^ and Ga_2_SeTe^44^ monolayers. In this work, we applied dipole correction in all calculations to account for this effect. However, it is worth noting that we found the intrinsic dipole to have minimal influence on the electronic properties of the metallic MoSH monolayer.

## 3. Results and discussion

**Fig. 1:**
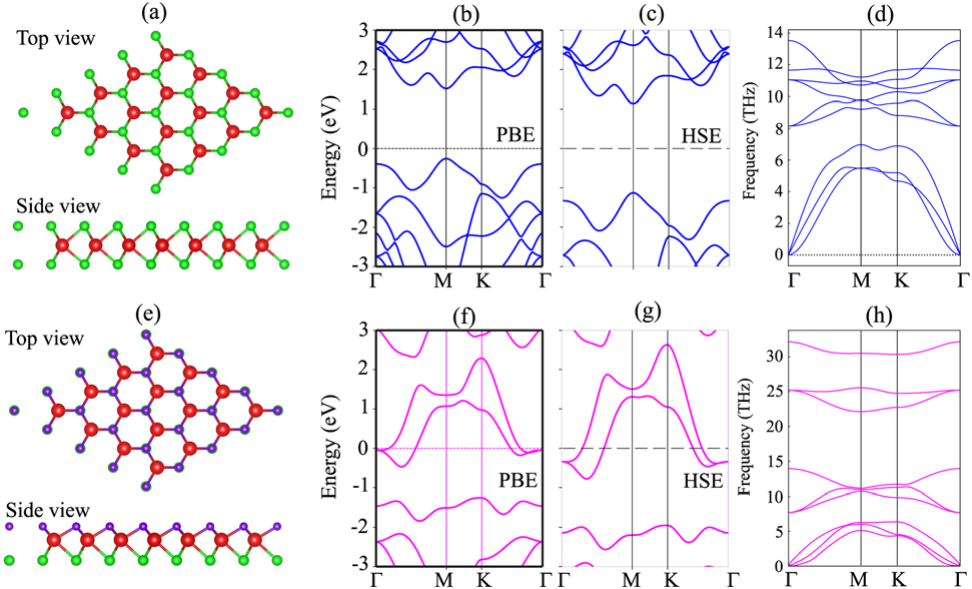
Atomic structures, band structures and phonon spectra of (a–d) MoS_2_ and (e–h) MoSH monolayers.

We first investigate the atomic structures and electronic properties of both MoSH and MoS_2_ monolayers. The atomic structures of MoS_2_ and MoSH monolayers are displayed in Fig. 1(a) and (e), respectively. Both the MoS_2_ and MoSH monolayers show the same atomic layered structure. In the MoS_2_ monolayer, one Mo atom is sandwiched between two S atoms on both sides, while in the MoSH monolayer, one Mo atom is sandwiched between one S and one H atom on each side. The lattice constants of MoS_2_ and MoSH monolayers are calculated to be 3.17 Å and 3.0 Å, which are in good agreement with the previous reports, both experimental and theoretical.^31,35^ The band structures of both MoS_2_ and Janus MoSH monolayers are calculated using PBE and HSE methods, which are represented in Fig. 1(b, c) and (f, g), respectively. We can find that the MoS_2_ monolayer displays a semiconducting behavior, whereas the Janus MoSH monolayer is a metal. The calculated band gap of the MoS_2_ monolayer is 1.77 eV for the PBE functional and 2.26 eV for the HSE functional. The PBE band gap is still smaller than that given from the experimental measurement of about 1.90 eV.^5^ The HSE functional can be used to obtain a more accurate band gap value for materials. One can see that the HSE band gap of the MoS_2_ monolayer is larger than that of the experimental measurement. Both the PBE and HSE methods predict similar behaviors in the formation of the metal–semiconductor MoSH@MoS_2_ heterostructure, as well as in the intrinsic properties of the metallic MoSH and semiconducting MoS_2_ monolayers. Therefore, we chose to use the PBE method in all our calculations due to the limitation of the computational cost. The phononic spectra of MoS_2_ and MoSH monolayers are displayed in Fig. 1(d) and (h). All the frequencies of these monolayers are positive at the Dirac *Γ* point, representing their dynamical stability.

**Fig. 2:**
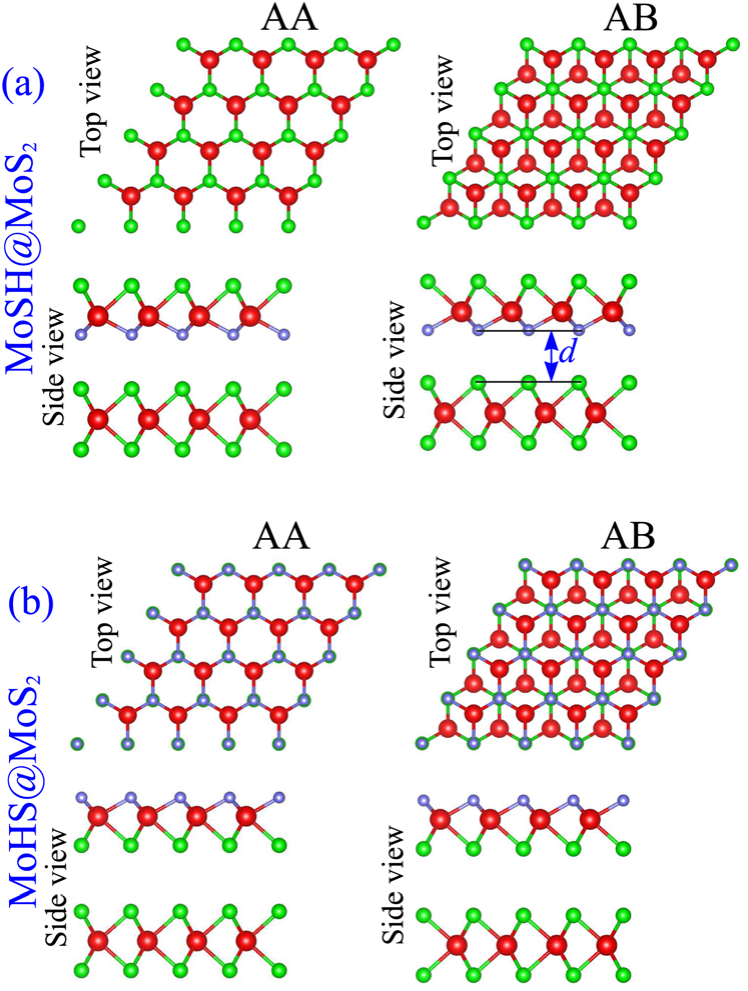
Atomic structures of (a) MoSH@MoS_2_ and (b) MoHS@MoS_2_ heterostructures for different stacking patterns of AA and AB.

We proceed with constructing the atomic structures of the metal–semiconductor heterojunction by combining metallic MoSH with semiconducting MoS_2_. The atomic configurations of the MoSH@MoS_2_ heterostructure are depicted in Fig. 2. The construction of the MoSH@MoS_2_ heterostructure involves maintaining the fixed lattice parameters of both MoSH and MoS_2_ monolayers. The resulting lattice constant of the MoSH@MoS_2_ heterostructure is determined to be 3.085 Å, representing the average of the lattice parameters of the individual monolayers. This indicates the presence of strain when the two materials are combined in the heterostructure. The lattice mismatch between the two monolayers in the heterostructure is calculated to be 2.75%, which is relatively small and has a negligible impact on the main properties of the materials. The formation of the MoSH@MoS_2_ heterostructure gives rise to the establishment of two distinct alignment configurations, namely MoSH@MoS_2_ and MoHS@MoS_2_ heterostructures. Each configuration consists of two different stacking patterns known as AA and AB stacking patterns, which are illustrated in Fig. 2(a) and (b). After the geometric optimization, the interlayer spacings *d* between two materials are obtained to be 2.79/2.86 Å for MoSH@MoS_2_ with the AB/AA stacking pattern and 3.53/3.54 Å for MoHS@MoS_2_ with the AB/AA stacking pattern. One can find that the interlayer spacing in the MoSH@MoS_2_ configuration is shorter than that in the MoHS@MoS_2_ configuration. This can be attributed to the weaker electronegativity of hydrogen atoms compared to sulfur atoms.

To determine the stability of the MoSH@MoS_2_ heterostructure, we further calculate the binding energy, which provides an estimate of the energy released when the heterostructure is formed, indicating the stability of the system. The binding energy (*E*_b_) can be calculated using the following formula:1
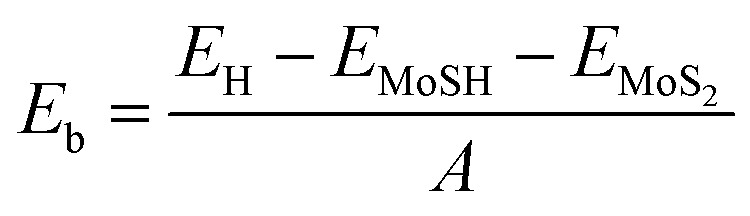
Here, *E*_H_ is the total energy of the MoSH@MoS_2_ heterostructure and *E*_MoSH_ and *E*_MoS_2__ are the total energies of the isolated MoSH and MoS_2_ monolayers, respectively. *A* stands for the surface area of the heterostructure. The calculated *E*_b_ for the MoSH@MoS_2_ heterostructure is −20.51 and −21.48 meV Å^−2^, respectively, for the AA and AB stacking patterns, while the calculated *E*b for the MoHS@MoS_2_ heterostructure is −17.61 and −17.94 meV Å^−2^, respectively, for the AA and AB stacking patterns. The minus “−” sign indicates that energy is released during the formation of the heterostructure, indicating the stability of the system. The magnitudes of the binding energies reflect the strength of the interaction between the MoSH and MoS_2_ layers. Higher absolute values of the binding energy indicate stronger binding and greater stability of the heterostructure. Based on the calculated binding energies, both the MoSH@MoS_2_ and MoHS@MoS_2_ heterostructures show favorable binding energies, indicating their stability. The AB stacking pattern of the MoSH@MoS_2_ heterostructure has the lowest binding energy, demonstrating that this stacking pattern is the most favorable stacking pattern.

**Fig. 3:**
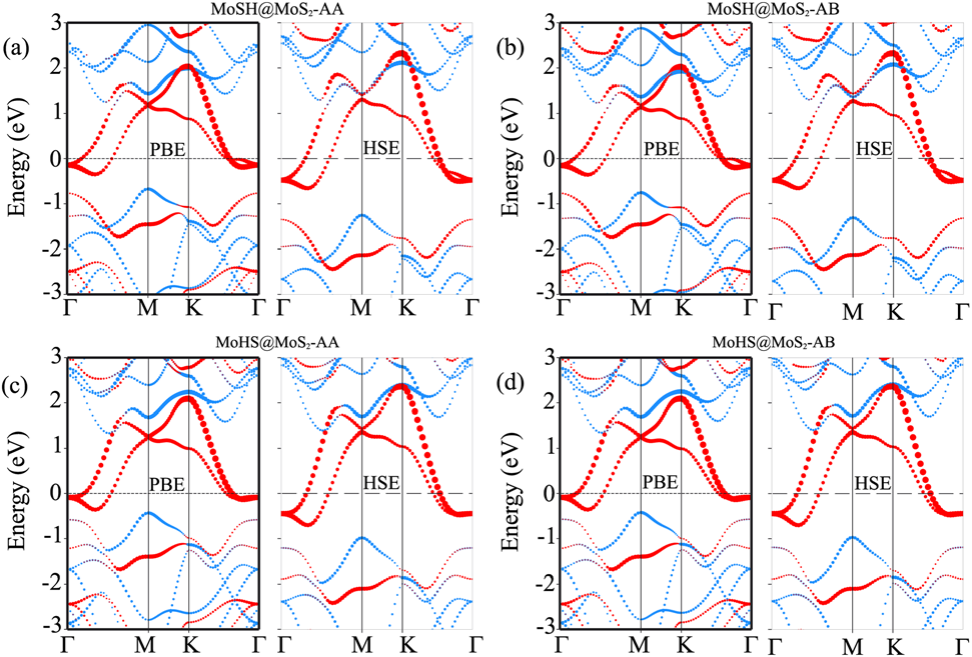
Projected band structures by PBE and HSE calculations of MoSH@MoS_2_ for (a) AA and (b) AB stacking patterns and MoHS@MoS_2_ for (c) AA and (d) AB stacking patterns. Red and green lines represent the contributions of MoSH and MoS_2_ layers, respectively.

We now calculate the projected band structures of both the MoSH@MoS_2_ and MoHS@MoS_2_ heterostructures for the AA and AB stacking patterns, as depicted in Fig. 3. Both the PBE and HSE functionals are used to predict the band structures of the heterostructure. Red and blue lines represent the contributions of Janus MoSH and MoS_2_ monolayers, respectively. We can find that all four stacking patterns show metallic characteristics. Furthermore, the combination between metallic MoSH and semiconducting MoS_2_ layers results in the generation of a metal/semiconductor heterojunction. This results in the possibility of forming either a Schottky or an ohmic contact, depending on the relative position of the band edges of the semiconducting MoS_2_ layer with respect to the Fermi level (*E*_F_). All the stacking patterns of the MoSH@MoS_2_ and MoHS@MoS_2_ heterostructures generate Schottky contact (ShC). The ShC in heterostructure is measured using the Schottky barrier height (SBH) which can be calculated using the following formula:

2 *Φ*_n_ = *E*_CBM_ − *E*_F_

and

3 *Φ*_p_ = *E*_F_ − *E*_VBM_

**Fig. 4:**
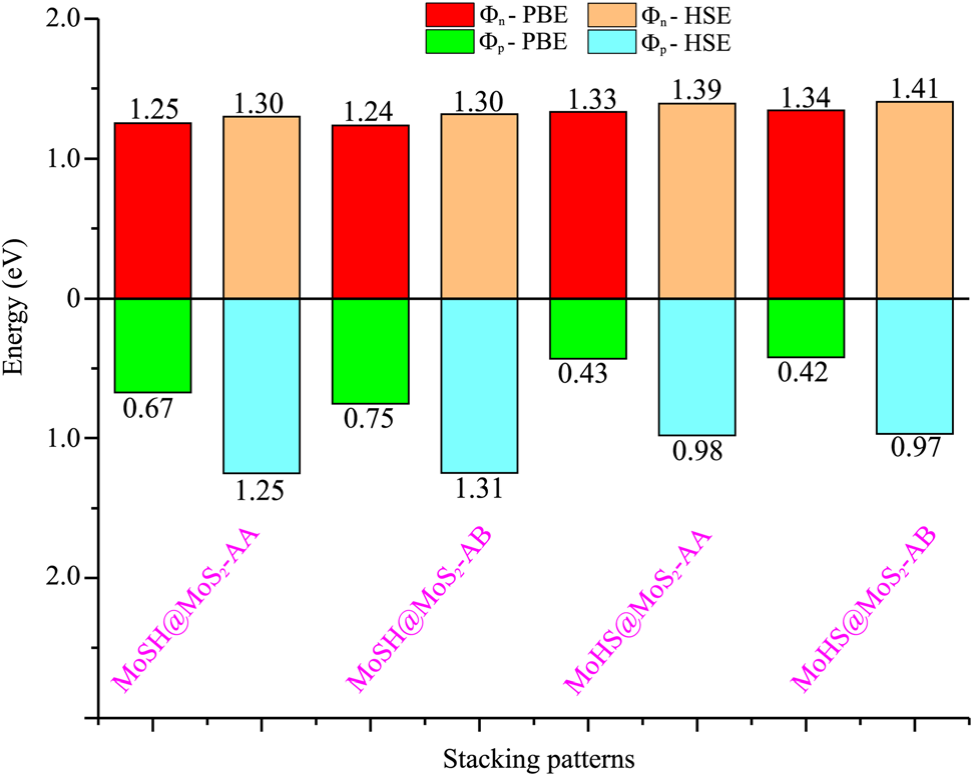
Calculated SBHs of the heterostructures between MoSH and MoS_2_ monolayers for different configurations using PBE and HSE functionals.

Here, E_CBM_ and *E*_VBM_ are the band edges of the semiconducting MoS_2_ layer. The SBH provides information about the energy barrier at the interface of the heterostructure, influencing charge transfer and device performance. By calculating the SBH, we can evaluate the electrical properties of the MoSH@MoS_2_ and MoHS@MoS_2_ heterostructures. The calculated SBHs of the heterostructures are depicted in Fig. 4. Indeed, all the stacking patterns of the heterostructures result in the formation of p-type ShC. Furthermore, among the various stacking patterns, the AB stacking pattern of the MoHS@MoS_2_ heterostructure exhibits the narrowest SBH of 0.42/0.97 eV for the PBE/HSE method, indicating a relatively easier charge transfer across the interface. On the other hand, the AB stacking pattern of the MoSH@MoS_2_ heterostructure demonstrates the highest SBH of 0.75/1.31 eV for PBE/HSE calculation, suggesting a larger energy barrier for charge transfer. These SBH values provide insights into the electronic properties and charge transport behavior at the metal/semiconductor interface in the MoSH@MoS_2_ and MoHS@MoS_2_ heterostructures.

**Fig. 5:**
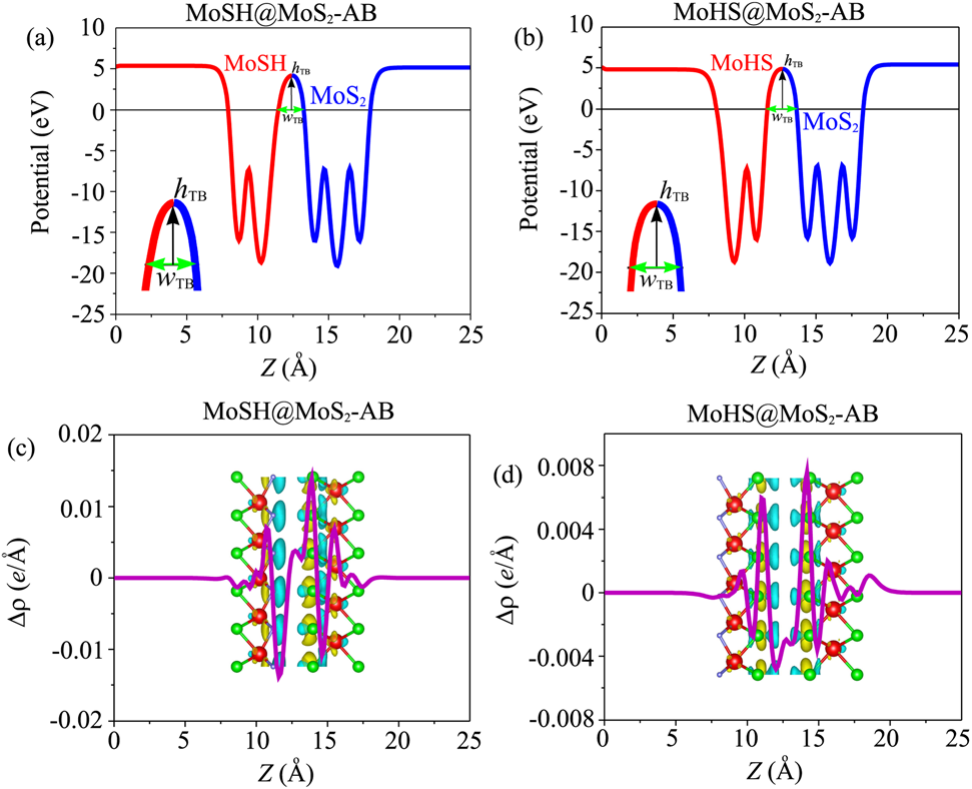
Electrostatic potential of (a) MoSH@MoS_2_ and (b) MoHS@MoS_2_ heterostructures for the AB stacking pattern. 2D planar-averaged charge density differences of (c) MoSH@MoS_2_ and (d) MoHS@MoS_2_ heterostructures for the AB stacking pattern. The yellow regions represent areas of charge accumulation, while the cyan regions indicate charge depletion.

Based on our previous discussion, it is evident that the AB stacking pattern is more favorable and stable than the AA stacking pattern for both the MoSH@MoS_2_ and MoHS@MoS_2_ heterostructures. The electrostatic potential and charge density differences (CDDs) of the MoSH@MoS_2_ and MoHS@MoS_2_ heterostructures for the AB stacking pattern are depicted in Fig. 5. Through the electrostatic potential of heterostructures, we can calculate the barrier height (*h*_TB_) and width (*w*_TB_) of the tunneling potential barrier. The calculated barrier height and width are 4.17 eV and 1.84 Å, respectively, for the MoSH@MoS_2_ heterostructure and are 4.87 eV and 2.06 Å, respectively, for the MoHS@MoS_2_ heterostructure. We further calculate the tunneling probability across the vdW interface, which can be obtained as follows:4
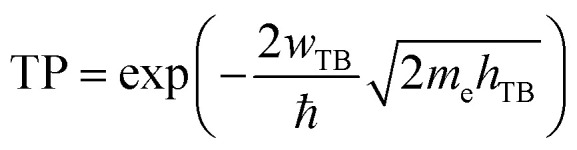
Here, *m*_e_ and ℏ are the free electron mass and reduced Planck constant, respectively. The tunneling probability for the MoSH@MoS_2_ heterostructure is found to be 3.39%, while for the MoHS@MoS_2_ heterostructure it is 4.10%. These values indicate a relatively low electron transparency across all the patterns of the MoSH@MoS_2_ heterostructures. The observed low tunneling probabilities imply that the electron transport across the MoSH@MoS_2_ heterostructures is relatively restricted. This information is crucial for understanding the electronic behavior and potential applications of these heterostructures in electronic devices, as it indicates the extent of electron transmission and the influence of the vdW interface on charge transport.

To gain a deeper understanding of the charge redistribution at the interface of the heterostructures, we plot the CDD of the heterostructure. The CDD represents the difference in charge density between the heterostructure and the isolated constituent layers, which can be visualized as follows:

5 Δ*ρ* = *ρ*_H_ − *ρ*_MoSH_ − *ρ*_MoS_2__

Here, *ρ*_H_, *ρ*_MoSH_ and *ρ*_MoS_2__ represent the charge densities of the heterostructure and isolated MoSH and MoS_2_ monolayers, respectively. From Fig. 5(c) and (d), it is evident that the charge depletion primarily occurs on the side of the sulfur and hydrogen layers of the MoSH layer. Conversely, the charge accumulation is mainly observed on the side of the sulfur layer of the MoS_2_ monolayer. These findings suggest a transfer of charge from the MoSH layer to the MoS_2_ layer, resulting in a redistribution of electrons at the interface. The charge depletion on the side of the MoSH layer and the charge accumulation on the side of the MoS_2_ layer reflect the charge transfer mechanism and the electronic interaction between the two layers in the heterostructure. Understanding the specific charge redistribution patterns provides valuable insights into the charge transfer dynamics and the electronic properties of the MoSH@MoS_2_ heterostructure. This information can be used to optimize the performance of electronic devices based on these heterostructures.

To assess the tunability of the heterostructure under an applied electric field, we investigate the response of the MoSH@MoS_2_ heterostructure to varying electric field strengths. By studying the response of the MoSH@MoS_2_ heterostructure to different electric field strengths, we can gain insights into its adaptability and potential for use in various electronic and optoelectronic applications. As previously discussed, the AB stacking pattern of the MoSH@MoS_2_ heterostructure exhibits the highest SBH among the different stacking patterns. This indicates a relatively larger energy barrier for charge transfer at the metal/semiconductor interface. On the other hand, the AB stacking pattern of the MoHS@MoS_2_ heterostructure has the narrowest SBH among the different stacking patterns. This suggests a relatively easier charge transfer across the interface due to a smaller energy barrier. The SBH is an important parameter that determines the efficiency and nature of charge transfer at the metal/semiconductor interface, influencing the overall performance and functionality of electronic devices based on these heterostructures. A lower SBH between the metal and semiconductor interface typically leads to an enhanced flow of current, which can improve the performance of electronic devices. This reduced barrier allows for easier electron or hole injection and facilitates efficient charge transport across the interface. Consequently, a lower SBH is often desired for achieving higher device performance, such as improved conductivity and enhanced device efficiency. The MoHS@MoS_2_ heterostructure, with its low SBH of 0.42 eV, indicates promising potential for high-performance Schottky devices.

**Fig. 6:**
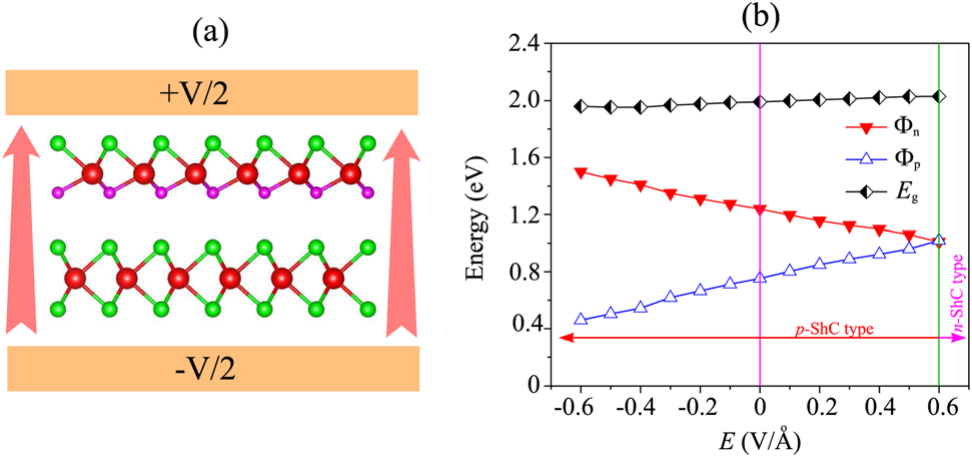
(a) Schematic illustration of the applied electric field and (b) the fluctuation of the Schottky barrier of the MoSH@MoS_2_ heterostructure as a function of the electric field.

We now turn to investigate the possibility of adjusting the SBH under an applied electric field of the heterostructure with the largest SBH. By subjecting this heterostructure to different electric field strengths, we aim to explore whether the SBH can be modified and controlled. The schematic model of the applied electric field to the MoSH@MoS_2_ heterostructure is depicted in Fig. 6(a). With the application of the electric field, the SBH of the MoSH@MoS_2_ heterostructure is changed, as depicted in Fig. 6(b). The negative electric field gives rise to a decrease in the *Φ*_p_, while the *Φ*_n_ increases correspondingly. The decrease in *Φ*_p_ suggests a reduction in the energy barrier for charge carrier injection from the metal to the semiconductor, potentially resulting in enhanced charge transport and an increased current flow. On the other hand, the positive electric field leads to an increase in the *Φ*_p_, while the *Φ*_n_ has decreased accordingly. At a positive electric field strength of +0.6 V Å^−1^, an interesting phenomenon occurs in the MoSH@MoS_2_ heterostructure: the narrowing of the Schottky barrier height (*Φ*_n_) relative to the Schottky barrier height (*Φ*_p_). This observation suggests the possibility of a transition from p-type Schottky contact (ShC) to n-type ShC. The narrowing of *Φ*_n_ compared to *Φ*_p_ indicates that the energy barrier for charge extraction from the semiconductor to the metal decreases more significantly than the energy barrier for charge injection from the metal to the semiconductor. As a result, the heterostructure's electrical behavior shifts from favoring the injection of holes (p-type) to the injection of electrons (n-type). This transition from p-type ShC to n-type ShC is significant as it opens up new avenues for device functionality and performance. It enables the heterostructure to exhibit ambipolar behavior, where both electron and hole transport can be efficiently controlled, leading to enhanced device versatility and potential applications in areas such as electronic logic circuits and optoelectronic devices.

**Fig. 7:**
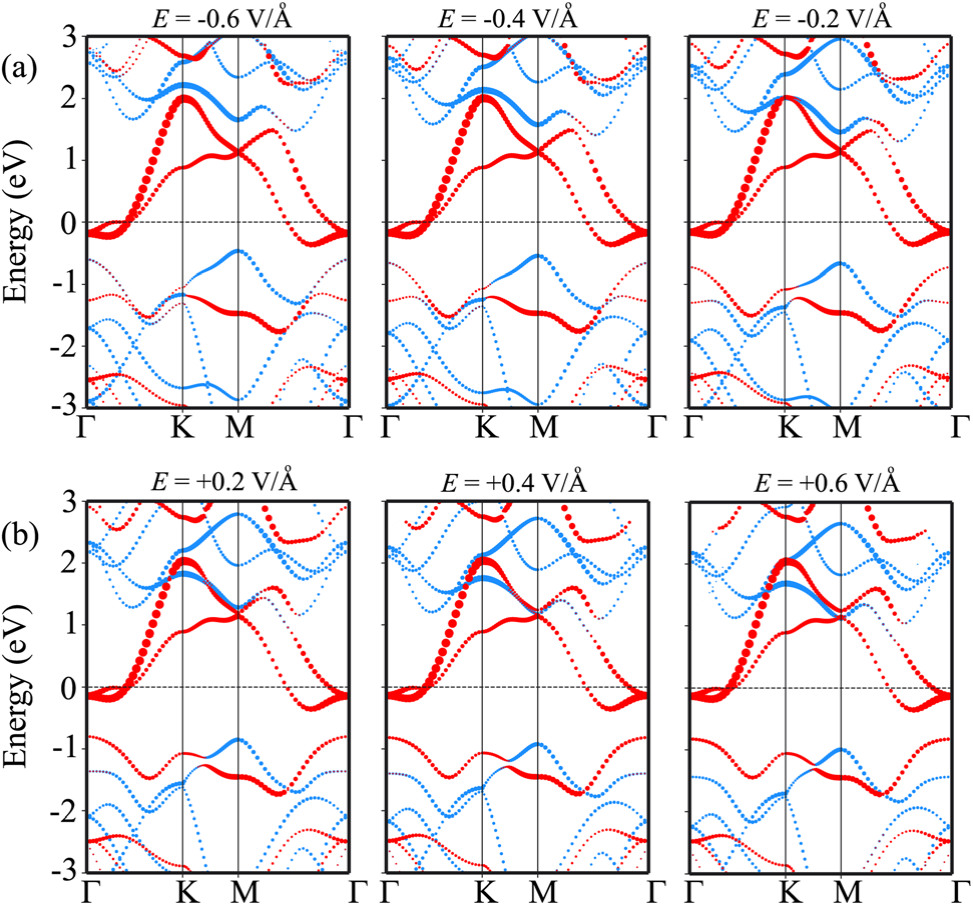
Projected band structures of the MoSH@MoS_2_ heterostructure for the AB pattern for different strengths of (a) negative and (b) positive electric fields. Red and green lines represent the contributions of MoSH and MoS_2_ layers, respectively.

**Fig. 8:**
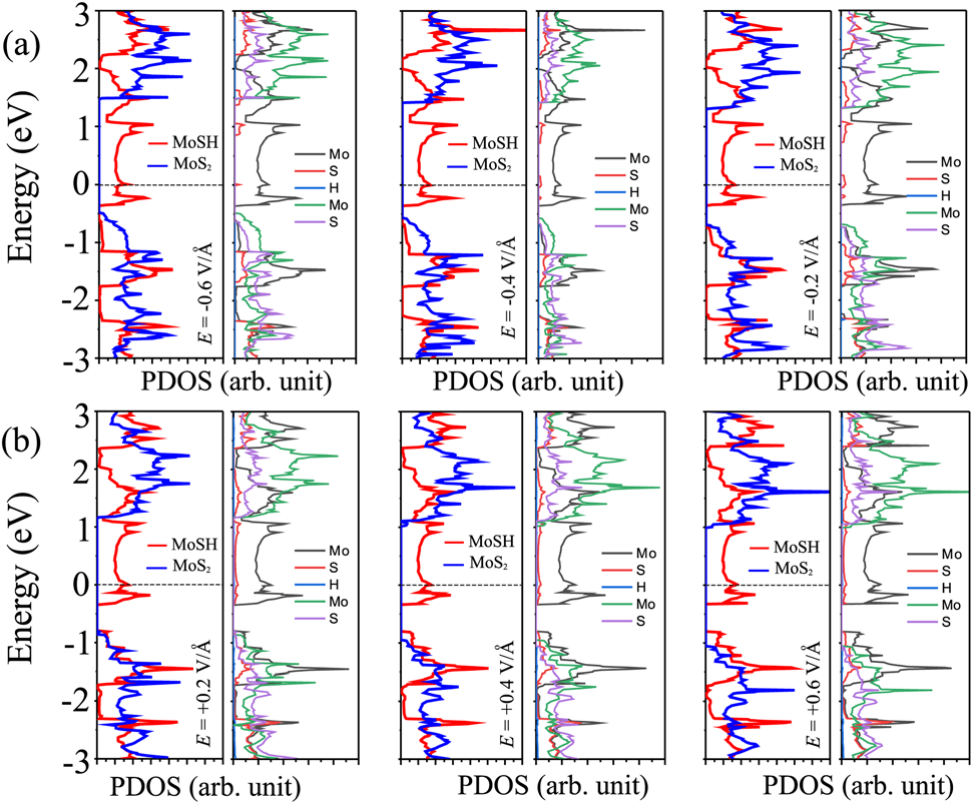
Projected density of states (PDOS) of Mo, S, and H atoms in the MoSH layer and Mo and S atoms in the MoS_2_ layer in the MoSH@MoS_2_ heterostructure under (a) applied negative and (b) positive electric fields.

In order to elucidate the underlying physical mechanism behind the changes in the SBH and contact types in the heterostructure, we plotted the projected band structure at various strengths of the electric field, as depicted in Fig. 7. Under the influence of a negative electric field, we observe distinct shifts in the band edges of the semiconducting MoS_2_ layers. Specifically, the VBM moves closer to the Fermi level, while the CBM moves further away from the Fermi level. These contrasting shifts in the band edges under a negative electric field contribute to the modification of the SBH and the resulting changes in the contact type in the heterostructure. Similarly, with the application of a positive electric field, the CBM of the semiconducting MoS_2_ shifts towards the Fermi level, while the VBM moves further away from the Fermi level. At a positive electric field of +0.6 V Å^−1^, the CBM of the semiconducting MoS_2_ layer is positioned closer to the Fermi level compared to its VBM. This arrangement indicates a transition from p-type ShC to n-type ShC. Furthermore, to gain a better understanding of the transition mechanism in the MoSH@MoS_2_ heterostructure, it is important to conduct either the crystal orbital Hamilton population (COHP) analysis ^45,46^ or the partial density of states (PDOS) analysis of all atoms, as depicted in Fig. 8. By analyzing the partial density of states (PDOS) of all atoms and each layer in the MoSH@MoS_2_ heterostructure, we can observe that the application of electric fields induces a shift in the band edges of the semiconducting MoS_2_ layer. Therefore, we can conclude that the applied electric field can be utilized to adjust both the SBH and contact types in the MoSH@MoS_2_ heterostructure. This capability to control the SBH and contact types through the application of an electric field provides opportunities for fine-tuning the electronic properties and performance of devices based on the MoSH@MoS_2_ heterostructure.

## 4. Conclusions

In conclusion, we have constructed a novel metal–semiconductor heterostructure between Janus MoSH and MoS_2_ monolayers and investigated its structures, electronic properties and the generation of Schottky contact using first-principles prediction. The effects of the electric field on controllable electronic properties and contact characteristics of MoSH@MoS_2_ heterostructures are also explored. Our findings show that the MoSH@MoS_2_ heterostructures exhibit p-type Schottky contact with different Schottky barrier heights, depending on the stacking configurations. The MoSH@MoS_2_ heterostructures possess low tunneling probabilities, indicating a relatively low electron transparency across all the patterns of the MoSH@MoS_2_ heterostructures. Furthermore, the applied electric field can be utilized to adjust both the SBH and contact types in the MoSH@MoS_2_ heterostructure. Our study sheds light on the potential applications of the MoSH@MoS_2_ heterostructure in electronic devices, demonstrating its tunability and versatility. This work contributes to the fundamental understanding of metal–semiconductor heterostructures and provides a foundation for further exploration and utilization of these materials in various technological applications.

## Data availability

The data that support the findings of this study are available from the corresponding author upon reasonable request.

## Conflicts of interest

There are no conflicts to declare.

## Supplementary Material
